# Spatio-temporal distribution and associated factors of home delivery in Ethiopia. Further multilevel and spatial analysis of Ethiopian demographic and health surveys 2005–2016

**DOI:** 10.1186/s12884-020-02986-w

**Published:** 2020-06-03

**Authors:** Zemenu Tadesse Tessema, Sofonyas Abebaw Tiruneh

**Affiliations:** 1 Department of Epidemiology and Biostatics, Institute of Public Health, College of Medicine and Health Sciences, University of Gondar, Gondar, Ethiopia; 2 Department of Public Health, College of Health Sciences, Debre Tabor University, P.O. Box. 272, Debre Tabor, Ethiopia

**Keywords:** Home delivery, EDHS, Spatial distribution, Ethiopia

## Abstract

**Background:**

Globally, between 2012 and 2017, 80% of live births occurred at health facilities assisted by skilled health personnel. In Ethiopia, in 2016 only 26% of live births attended by skilled health personal. This study aimed to assess the spatial patterns and associated factors of home delivery in Ethiopia using 2005, 2011, and 2016 Ethiopian Demographic and Health Surveys.

**Methods:**

A total of 33,482 women who gave live birth in the 5 years preceding each survey were included for this study. ArcGIS version 10.7 software was used to visualize the spatial distribution of home delivery. The Bernoulli model was applied using Kilduff SaTScan version 9.6 software to identify significant purely spatial clusters for home delivery in Ethiopia. A multilevel logistic regression model was fitted to identify factors associated with home delivery. A *p*-value < 0.05 was taken to declare statistically significant predictors.

**Results:**

Home delivery was declined from 94.78% in 2005, 90.05% in 2011, and 73.44% in 2016 in Ethiopia. Among the three surveys, consistently high clustering of home delivery was observed in Amhara and Southern Nations Nationalities and People’s Regions (SNNPR) of Ethiopia. In spatial scan statistics analysis, a total of 128 clusters (RR = 1.04, *P*-value < 0.001) in 2005, and 90 clusters (RR = 1.11, *P*-value < 0.001) in 2011, and 55 clusters (RR = 1.29, *P*-value < 0.001) in 2016 significant primary clusters were identified. Educational status of women and husband, religion, distance to the health facility, mobile access, antenatal care visit, birth order, parity, wealth index, residence, and Region were statistically significant predictors of home delivery.

**Conclusion:**

The spatial distribution of home delivery among the three consecutive surveys were non-random in Ethiopia. Educational status of women and husband, religion, distance to the health facility, wealth index, residence, parity, mobile access, Region, and antenatal care visit were statistically significant predictors of home delivery in Ethiopia. Therefore, an intervention needs to improve the coverage of antenatal care visit, and health care facilities. Ministry of health and other stakeholders should give special attention to women living in Amhara and SNNPR states of Ethiopia.

## Background

Maternal mortality reduction remains a priority agenda under goal three in the UN Sustainable Development Goals (SDGs) through 2030 [1]. Worldwide, about 295,000 maternal deaths occurred in 2017 which is 38% of reduction since the year 2000 an average reduction of just under 3% per year. Even though a significant decline in maternal mortality in the last 25 years, still maternal mortality is unacceptably high [2]. Every day, about 810 women died from preventable and related causes to pregnancy and childbirth, which is the vast majority of these deaths (94%) that occurred in low-resource setting countries [3].

Maternal mortality in Ethiopia fell from 1250 deaths per 100,000 livebirths in 1990 to 353 deaths per 100,000 livebirths in 2015, declined by 71.8% which is below the target of Millennium Development Goals (MDGs) related to maternal mortality [4, 5]. Sustainable Development Goal (SDG) goal 3 calls for the ambition of maternal mortality ratio reduction less than 70 per 100,000 live births between 2016 to 2030 [6].

About 73% of all maternal deaths were due to direct obstetric cases and deaths due to indirect causes accounted for 27·5% of all maternal deaths [7]. Nearly one-quarter of maternal death occurred in the antepartum period, another quarter occurred in the intrapartum and immediate postpartum periods, one-third occurred in the subacute and delayed postpartum periods, and 12% occurred in the late postpartum period [8].

Globally, between 2012 and 2017 only 80% of live births occurred at health facilities assisted by skilled health personnel. However, only 59% of the live births were attended by skilled health personal in the Sub-Saharan Africa region, where maternal mortality is the highest [9]. Even though, skilled childbirth before, during, and after can save the lives of women, still in Ethiopia, 94.5% in 2000, 93.1% in 2005, 87.9% in 2011, and 73.6% in 2016 birth attended at home which is unacceptable high [10, 11].

In Ethiopia, several studies evidenced that, women’s low attainment of educational status, cultural factors, communal factors, limited access to health facilities, poor quality of care, lack of transportation, and poor wealth status were the significant factors that lead to low maternal health services utilization [10, 12–14]. So far different studies in Ethiopia done to identify the factors for the choice of place of delivery [15–17]. The spatial distribution of home delivery was unclear in Regions of Ethiopia. Identifying the spatial distribution of home delivery in Ethiopia can help health planners and policymakers for specific interventions to decrease home delivery. This study aimed to assess the spatial distributions of home delivery and associated factors of home delivery in Ethiopia using 2005, 2011, and 2016 Ethiopian Demographic and Health Survey (EDHS) datasets.

## Methods

### Study design, period and setting

A cross-sectional survey study design was conducted in Ethiopia using 2005, 2011, and 2016 EDHS. Ethiopia is located in the Horn of Africa and has 9 Regional states namely Tigray, Afar, Amhara, Oromia, Somali, Benishangul Gumuz, Southern Nations, Nationalities and People’s Region (SNNPR), Gambella, and Harari Regional states and two administrative cities namely Addis Ababa and Dire Dawa.

### Source and study population

The source population was all reproductive age group women (15–49) in Ethiopia. The study population was women who gave birth in the last 5 years preceding each survey for the recent birth.

### Sample size and sampling procedure

A total of 33,482 women (10,721 women in 2005, 11,872 women in 2011, and 10,889 women in 2016) were included in this study. Weighted values were used to restore the representativeness of the sample data. Sample weights were calculated in each children’s record (KR) EDHS datasets. The survey covered all nine regions and the two city administrations of Ethiopia. Participants were selected based on a stratified two-stage cluster sampling technique in each survey year (2005, 2011, and 2016). After excluding clusters with zero coordinates and no birth records, a total of 527 clusters in 2005, 571 clusters in 2011, and 622 clusters in 2016 were included. The detailed sampling procedure was available in each EDHS reports from Measure DHS website (www.dhsprogram.com).

### Data collection tools and procedures

The data obtained from the Demographic and Health Surveys (DHS) Program by requesting for this work and accessed www.dhsprogram.com website. Ethiopian Demographic and Health Survey data collected by two-stage stratified sampling. Each region of the country was stratified into urban and rural areas.

### Variables

#### Outcome variable

The outcome variable taken as binary response woman gave birth at home and others home coded as “1” which is home delivery, and women gave birth different governmental health facilities, private health facilities, and non-governmental health facilities coded as “0” which is health facility delivery.

#### Independent variables

From the EDHS dataset all sociodemographic and obstetric characteristics (individual and community level) taken as independent variables in the three-consecutive survey.

### Data management and analysis

The data cleaned by STATA version 14.1 software and Microsoft excel. Sample weighting was done before further analysis.

### Spatial autocorrelation

We used Arc GIS 10.7 software for spatial autocorrelation and detection of hot spot areas. Spatial autocorrelation (Global Moran’s I) statistic measure was used to assess whether home delivery was dispersed, clustered, or randomly distributed in Ethiopia. Moran’s I values close to − 1 indicates dispersed home delivery, close to + 1 indicates clustered, and if Moran’s I value zero indicates randomly distributed [18].

### Hot spot analysis

The proportion of home delivery in each cluster was taken as an input to analyze the hotspot analysis. Hot Spot Analysis (Getis-Ord Gi* statistic) of the z-scores and significant *p*-values tells the features with either hot spot or cold spot values for the clusters spatially. The hot spot areas indicated that there was a high proportion of home delivery and the cold spot indicated that there was a low proportion of home delivery. The maximum peak distance in which spatial dependency pronounced at 151.37 Km in 2016, 148.13 Km in 2011, and 193.9 km in 2005 EDHSs.

### Spatial interpolation

The spatial interpolation technique is used to predict home delivery for unsampled areas based on sampled clusters. For the prediction of unsampled clusters, we used geostatistical ordinary Kriging spatial interpolation technique using ArcGIS 10.7 software. In each EDHS survey year, home delivery is known for all enumeration areas, but home delivery for other unselected locations in Ethiopia is also of interest.

### Spatial scan statistics

We employed Bernoulli based model spatial scan statistics to determine the geographical locations of statistically significant clusters for home delivery using Kuldorff’s SaTScan version 9.6 software [19]. The scanning window that moves across the study area in which women gave birth at home were taken as cases and those women who gave birth at health facility taken as controls to fit the Bernoulli model. The default maximum spatial cluster size of < 50% of the population was used as an upper limit, allowing both small and large clusters to be detected, and ignored clusters that contained more than the maximum limit with the circular shape of the window. Most likely clusters were identified using *p*-values and likelihood ratio tests based on 999 Monte Carlo replications.

### Model building

We fit four models, the null model without predictors, the model I with only individual-level variables, model II with only community-level variables, and model III both individual-level and community-level variables. These models were fitted using a STATA command ***xtmelogit*.** For model comparison, we used the Log-Likelihood Ratio (LLR). The highest log-likelihood wins the best-fitted model.

### Parameter estimation methods

In the multilevel multivariable logistic regression model, fixed effect estimates measure the association between the odds of home delivery of individual and community level factors with a 95% confidence interval. Univariate analysis was carried out. A *P*-value less than or equal to 0.25 was taken as the candidate variable for the multivariable analysis. Adjusted Odds Ratio (AOR) with 95% CI and P-value less than 0.05 were reported as a significant factor that affects home delivery. Multicollinearity was checked using the Variance Inflation Factor (VIF). VIF less than 10% was taken as no multicollinearity.

The random effect measures variation of home delivery across clusters expressed by Intraclass Correlation (ICC) which quantifies the degree of heterogeneity of home delivery between clusters, Percentage Change in Variance (PCV) the proportion of the total observed individual variation of home delivery that is attributable to between cluster variations, and Median Odds Ratio (MOR) median value of the odds ratio between the cluster at high-risk home delivery and cluster at lower risk of home delivery when randomly picking out two clusters.

## Results

### Background characteristics of individual women

**Fig. 1:**
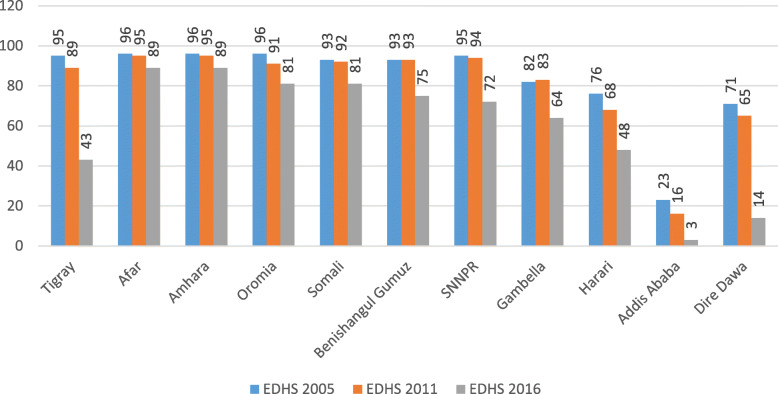
Trends in Home Delivery Overtime across the regions, Ethiopia in EDHS 2005, 2011, and 2016

A total of 33,482 women (10,721 in 2005, 11,872 in 2011, and 10, 889 in 2016) were included for this study. Overall, 94.78, 90.05, and 73.44% of women gave birth at home in 2005, 2011, and 2016 EDHSs respectively. From the three consecutive surveys, more than 60% of the mothers were in the age group of 20–34 years and had the same mean ± SD age of 29 ± 6.6 years. Among the three surveys, a significant number (48%) of the female household head was observed in the 2011 EDHS survey. All most all (> 90%), of the women were married in 5 years preceding the survey in three consecutive surveys. The educational status of the women was 79, 69, and 66% were unable to read and write in each survey year respectively. As well, 31, 47, and 56% of women were had not any work in the consecutive surveys respectively. In the EDHS 2016 survey, personal mobile and health insurance status were interviewed but not in EDHS 2005 and 2011. In 5 years, preceding the survey 16.4% of women were had personal mobile and 3.46% of women were insured for health insurance (Table 1). Despite the high prevalence of home delivery in Ethiopia, all regions registered in the decreasing trend from 2005 to 2016 (Fig. 1).

**Table 1:** Sociodemographic characteristics of women who had a live birth in the five years preceding the survey from EDHS 2005, 2011, and 2016 in Ethiopia

Characteristics		EDHS years		
		2005	2011	2016
		Frequency (%)	Frequency (%)	Frequency (%)
Women age	< 20 Years	1315 (12.26)	1132 (9.53)	851 (7.81)
	20–34 years	6655 (62.07)	7861 (66.22)	7337 (67.38)
	35–49 Years	2752 (25.67)	2879 (24.25)	2701 (24.80)
	Mean ± SD	29.01 ± 6.94	29.04 ± 6.63	29.57 ± 6.60
Household head	Male	9558 (89.15)	6168 (51.95)	9371 (86.06)
	Female	1163 (10.85)	5704 (48.05)	1518 (13.94)
Marital status	Not having partner	488 (4.50)	721 (6.07) 6.07	681 (6.25)
	Had partner	10,233 (95.50)	11,151 (93.93) 100.00	10,208 (93.75)
	Total	10,721 (100)	11, 872 (100)	10,889 (100)
Religion	Orthodox	4543 (42.37)	4519 (38.06) 38.06	3718 (34.14)
	Muslim	3752 (35.00)	4214 (35.49) 73.56	4519 (41.50)
	Protestant	2138 (19.91)	2758 (23.23) 96.78	2297 (21.09)
	Others	286 (2.68)	382 (3.22) 100.00	355 (3.26)
Residence	Urban	760 (7.09)	1528 (12.87) 12.87	1213 (11.14)
	Rural	9961 (92.91)	10,344 (87.13)	9676 (88.86)
Region	Tigray	684 (6.39)	753 (6.34)	701 (6.44)
	Afar	104 (0.97)	121 (1.02)	114 (1.05)
	Amhara	2571 (23.99)	2656 (22.37) 29.74	2041 (18.74)
	Oromia	4255 (39.69)	5014 (42.23) 71.97	4813 (44.20)
	Somalia	455 (4.24)	365 (3.07)	508 (4.66)
	Benishangul Gumuz	83(.78)	140 (1.18)	120 (1.11)
	SNNP	2338 (21.81)	2494 (21.01)	2250 (20.67)
	Gambela	29(.27)	40 (0.34)	27 (0.24)
	Harari	20(.19)	29 (0.24)	26 (0.24)
	Addis Ababa	141 (1.32)	222 (1.87)	243 (2.33)
	Dire Dawa	36(.34)	39 (0.33)	46 (0.42)
Women education	Unable to read and write	8502 (79.31)	8227 (69.30) 69.30	7201 (66.13)
	Primary education	1772 (16.53)	3211 (27.05)	2904 (26.67)
	Secondary education	409 (3.79)	266 (2.24)	510 (4.68)
	Higher education	40 (.38)	168 (1.42)	274 (2.52)
Husband education	Unable to read and write	6308 (59.03)	5966 (50.60)	5018 (48.57)
	Primary education	3245 (30.37)	4867 (41.26)	4051 (39.21)
	Secondary education	1023 (9.58)	584 (4.95) 6.81	790 (7.65)
	Higher education	109 (1.01)	376 (3.19)	471 (4.56)
Women occupation	Not working	7623 (30.71)	5597 (47.14)	6057 (55.62)
	Working	3098 (28.89)	6275 (52.86)	4832 (44.38)
Husband occupation	Not working	215 (2.00)	158 (1.33)	6199 (56.93)
	Working	10,506 (98.00)	11,714 (98.67)	4690 (43.07)
Distance to health facility	Big problem	6597 (60.58)	8956 (75.43)	6597 (60.58)
	Not a big problem	4292 (39.42)	2916 (24.57)	4292 (39.42)
Media exposure	No media exposure	7347 (68.41)	8956 (75.43)	7347 (68.41)
	Has media exposure	3392 (31.59)	2916 (24.57)	3392 (31.59)
Had mobile	No	NA	NA	9103 (83.60) 83.60
	Yes	NA	NA	1786 (16.40)
Insurance	Not insured	NA	NA	10,512 (96.54)
	Insured	NA	NA	377 (3.46)
Had ANC	No	5041 (71.86)	4543 (57.45)	2792 (37.42)
	Yes	1973 (28.14)	3365 (42.55)	4670 (62.58)
	Total	7014 (100)	7908 (1000)	7462 (100)
Birth order	1	2030 (18.65)	2261 (19.05)	2030 (18.65)
	2–4	4660 (42.80)	5179 (43.62)	4661 (42.80)
	≥5	4197 (35.55)	4432 (37.33)	4198 (38.55)
Parity	≤ 2	3135 (28.80)	3469 (29.22)	3136 (28.80)
	2–5	4319 (39.67)	4710 (39.67)	4319 (39.67)
	≥ 5^+^	3433 (31.53)	3693 (31.11)	3434 (31.53)
Wealth index	Poor	5091 (46.67)	5368 (45.22)	5091 (46.76)
	Middle	2243 (20.60)	2437 (20.53)	2243 (20.60)
	Richer	3554 (32.64)	4067 (34.26)	3555 (32.64)
Place of delivery	Health institution	560 (5.22)	1180 (9.95)	2891 (26.56)
	Home	10,161 (94.78)	10.691 (90.05)	7997 (73.44)
Total		**10,721 (100)**	**11,872 (100)**	**10,889 (100)**

NB: NA = Indicates data not available in that EDHS year

### Characteristics of the cluster

The unit of analysis for the community factors on home delivery was clusters. In EDHS 2016, 645 clusters were selected; from these 643 clusters were eligible in which the women give birth preceding 5 years the survey. Among the total number of clusters, 69% were rural in residence and almost half (49%) of the clusters were had a big problem accessing any health institution. Regarding the aggregate community, ANC utilization rate half of the clusters were had low community utilization. From the total of clusters, half of them were had low community women educational attainment and high community poverty status (Table 2).

**Table 2:** Characteristics of clusters in EDHS 2016

Community-level characteristics		Frequency	Percent (%)
Residence	Rural	441	68.68
	Urban	202	31.32
Region	Tigray	63	9.80
	Afar	53	8.24
	Amhara	71	11.04
	Oromia	74	11.51
	Somali	67	10.42
	Benishangul	50	7.78
	SNNPR	71	11.04
	Gambela	50	7.78
	Harari	44	6.84
	Addis Ababa	56	8.71
	Dire Dawa	44	6.84
Community ANC utilization rate	Low	320	49.77
	High	323	50.23
Community distance to the health facility	Big problem	314	48.83
	Not a big problem	329	51.17
Community media exposure	Low	321	49.92
	High	322	50.08
Community poverty status	High	319	49.61
	Low	324	50.39
Community-women educational attainment	Low	318	49.46
	High	325	50.54
Total		**643**	**100**

### Spatio-temporal distribution of home delivery in Ethiopia

The spatial distribution of home delivery in Ethiopia was non-random among the three consecutive surveys. The global Moran’s I value was 0.12 (*P*-value < 0.001) in 2005, 0.59 (*P*-value < 0.001) in 2011, and 0.44 (*P*-value < 0.001) in 2016 Ethiopian Demographic and health surveys.

### Hot spot analysis of the three surveys

**Fig. 2:**
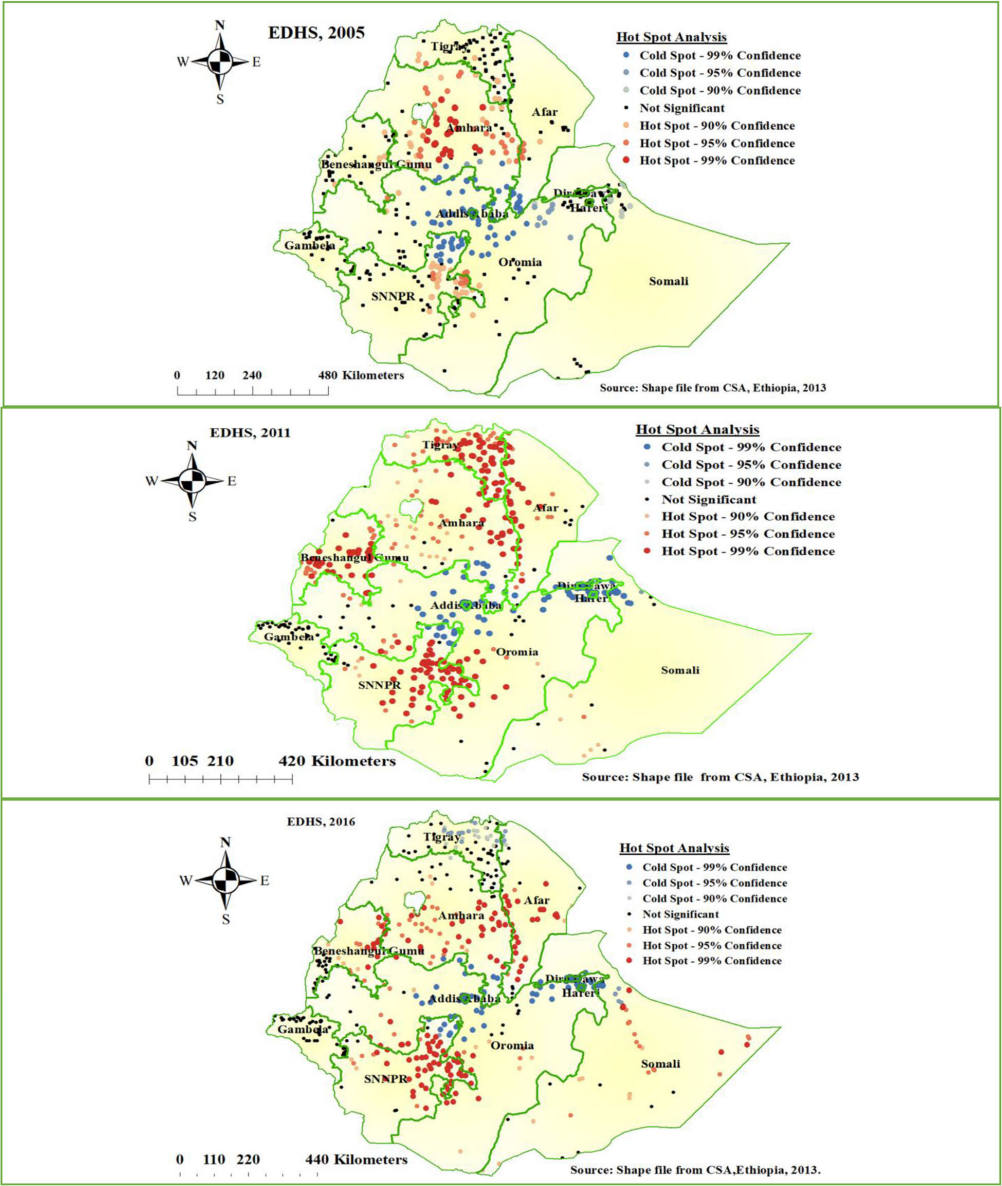
Hot spot analysis of home delivery in Ethiopia, EDHS 2005 to 2016 (Source: Shape file from Ethiopia Central Statistical Agency (CSA), 2013)

The spatial distribution of home delivery in Ethiopia was different in the three survey years. In EDHS 2005, a high proportion of home delivery was detected mainly at Amhara and SNNPR regional states of Ethiopia. In EDHS 2011, high clustering of home delivery detected in most parts of Tigray, Amhara, Afar, Benishangul and SNNPR, and western part of the Oromia region of Ethiopia. Furthermore, in EDHS 2016 high proportion of home delivery detected in the southern part of Afar, Southeastern part of Amhara, SNNPR, Benishangul Gumuz, and Somali region of Ethiopia (Fig. 2).

### Spatial scan statistics analysis of the three survey years

**Fig. 3:**
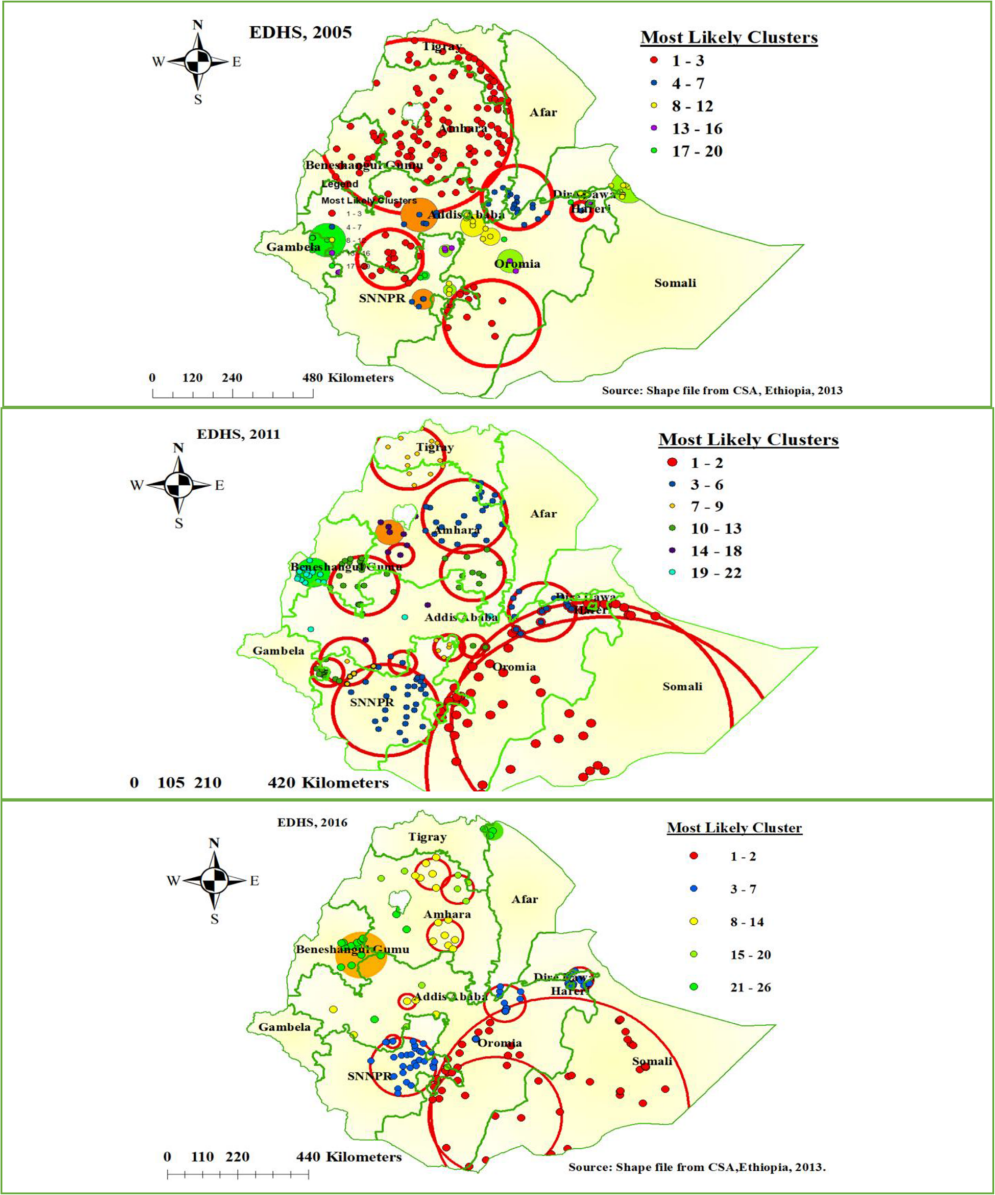
SaTScan scan statistics analysis of home delivery in Ethiopia, EDHS 2005 to 2016 (Source: Shape file from Ethiopia Central Statistical Agency (CSA), 2013)

As shown in Fig. 3 below, the red window indicates the identified significant clusters inside the window. In spatial scan statistics, a total of 158 most likely clusters were identified in EDHS 2005 survey. The most likely clusters of home delivery were detected in most parts of Amhara, southwestern part of Tigray, SNNPR, and Eastern part of the Benishangul Gumuz region of Ethiopia. Among the most likely clusters, 128 of them were primary clusters which are located at 11.586460 N and 37.367962 E with 290.16 km radius. In EDHS 2005, mothers live in the primary cluster were 4% more likely to give birth at home than outside the window (Relative risk (RR) = 1.04 and Log-Likelihood ratio (LLR) = 45.75, *P*-value < 0.001).

In EDHS 2011, a total of 127 most likely clusters were identified in spatial scan statistics which is located at Southeastern Oromia and Sothern part of the Somali region of Ethiopia. Among the most likely cluster, 90 of them were primary clusters located at 5.842888 N and 42.105068 E with 396.38 km radius. Mothers live in the primary cluster were 11% more likely to deliver at home as compared to outside the window (RR = 1.11, LLR = 161.45, *P*-value < 0.001).

Furthermore, in the 2016 EDHS survey, 70 most likely clusters were detected spatially. The most likely clusters of home delivery were detected in southeastern Oromia and the western Somali region of Ethiopia. From the most likely clusters, 55 of them were primary clusters located at 5.330795 N and 41.837597 E with 400.35 km radius. Mothers who live in the primary clusters were 29% more likely to give birth at home as compared to outside the window (RR = 1.29, LLR = 210.89, *P*-value < 0.001) (Fig. 3).

### Prevalence of home delivery in Ethiopia in the three EDHS surveys

**Fig. 4:**
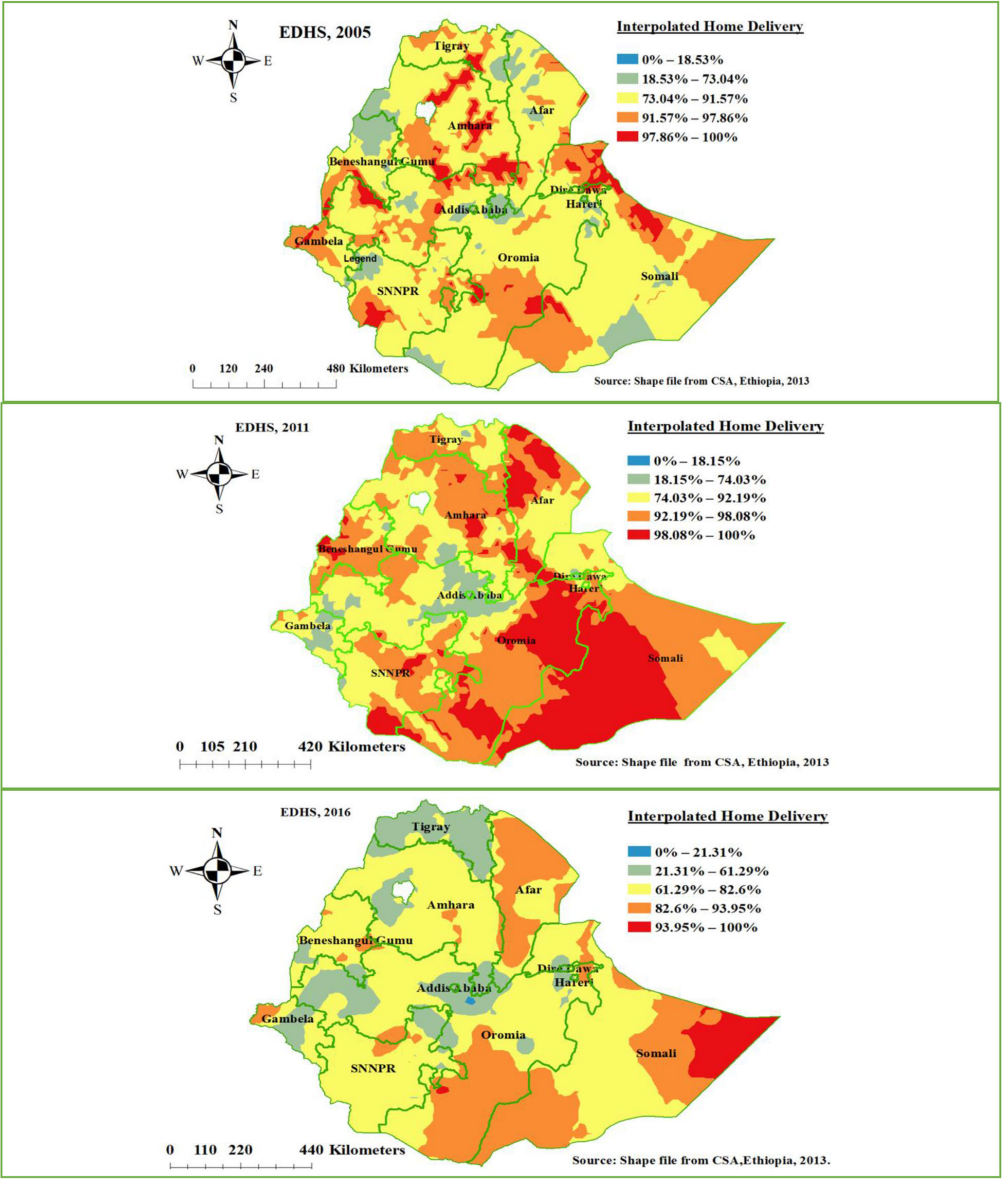
Ordinary Kriging interpolation of home delivery in Ethiopia, EDHS 2005 to 2016 (Source: Shape file from Ethiopia Central Statistical Agency (CSA), 2013)

Based on geostatistical Kriging analysis, in 2005 EDHS exclusively Dira Dawa, Harari, and Addis Ababa had a prevalence of home delivery less than 73%. In 2011 EDHS survey still, there is no significant difference in-home delivery reduction compared to 2005 EDHS across the region of Ethiopia. In the EDHS 2016 survey, Tigray, Addis Ababa, some parts of Oromia, Dire Dawa, and Gambela region significantly decreased home delivery in Ethiopia as compared to the other region (Fig. 4).

### Multilevel analysis (random effect analysis)

A Home delivery prevalence rate was not similarly distributed across the communities. About 67.32% of the variance in the odds of home delivery in women could be attributed to community-level factors, as calculated by the ICC based on estimated intercept component variance and the variation was statistically significant (*p*-value < 0.001). After adjusting for individual-level and community-level factors, the variation in-home delivery across communities remained statistically significant. About 89% of the odds of home delivery variation across communities was observed in the full model (Model 4). Moreover, the MOR indicated that home delivery was attributed to community-level factors. The MOR for home delivery was 12.17 in the empty model (Model 1); this showed that there was variation between communities (clustering) since MOR was 12.17 times higher than the reference (MOR = 1). The unexplained community variation in-home delivery decreased to MOR of 2.54 when all factors were added to the null model (empty model). This indicates that when all factors are included, the effect of clustering is still statistically significant in the full model.

### Individual-level predictors for home delivery

This study evidenced that on multivariable multilevel logistic regression analysis, individual-level factors such as religion, ANC visit, wealth index, birth order, parity, distance from the health facility, insurance, and mobile access were statistically significant factors for home delivery.

Keeping all individual and community level factors constant, the odds of giving birth at home for protestant in religion followers was 1.62 times higher than orthodox followers (AOR = 1.62, (95% CI, 1.15, 2.28)). Mothers who had personal mobile were 40% less likely gave birth at home than those who had no personal mobile (AOR = 0.60, (95%CI, 0.48, 0.76)). Women who had at least one ANC visit at the health facility were 82% less likely to deliver at home than those who had no ANC visit (AOR = 0.18, (95% CI, 0.14, 0.22)). The odds of giving birth at home among women who had 2–5 and more than five birth order were 78 and 87% than birth order less than 2 respectively (AOR = 1.78, (95% CI, 1.36, 2.34), AOR = 1.87, (95%CI, 1.27, 2.76)). Multiparous women (para 2–5) had high odds of giving birth at home by 37% as compared to para II women (AOR = 1.37, 95% CI, 1.08, 1.74)). Furthermore, mothers rich in their wealth were 22% less likely to deliver at home as compared to poor wealth status (AOR = 0.78, (95%CI, 0.61, 0.99)).

### Community-level predictors for home delivery

In the multivariable multilevel logistic regression model residence, region, community ANC utilization rate, community women education, and distance to any health institution were significantly associated with community-level factors for place delivery.

Keeping all individual and community level factors constant, women in rural clusters were three times more likely to give birth at home than women in urban clusters (AOR = 2.92, (95%, 1.99, 4.29)). Regarding regions, women live in Afar, Amhara, and Somali had high odds of giving birth at home as compared to Addis Ababa (AOR = 11.03, (95% CI, 4.29, 28.41), AOR = 6.39, (95% CI, 2.60, 15.70), AOR = 6.02, (95% CI < 2.41, 15.06)). Women live in Tigray and Dire Dawa were no significant difference in the place of delivery as compared to Addis Ababa (AOR = 1.46, (95% CI, 0.59, 3.60), AOR = 2.01, (95% CI, 0.77, 5.20)). Women who live in a high community ANC utilization were 50% less likely to give birth at home than in low community ANC utilization in 5 years preceding the survey (AOR =0.50, (95% CI, 0.39, 0.65)). Women in a cluster had no problem accessing any health institution were less likely to deliver at home by 29% those women had a problem accessing health facilities (AOR = 0.69, (95%CI, 0.53, 0.90)). Furthermore, high women education status in the cluster (community) was 28% less likely deliver at home than low women education attainment at the cluster in 5 years preceding the survey (AOR = 0.78, (95% CI, 0.60, 0.99)) (Table 3).

**Table 3:** Multivariable multilevel logistic regression analysis of both individual and community-level factors associated with Home Delivery in Ethiopia, EDHS 2016

Individual and community-level variables	Models			
	Null model	Model I	Model II	Model III
	AOR (95%CI)	AOR (95%CI)	AOR (95%CI)	AOR (95%CI)
Mother’s age				
< 20 Years		1		1
20–34 years		0.79 (0.59, 1.05)		0.90 (0.67, 1.20)
35–49 Years		0.70 (0.49, 1.01)		0.91 (0.63, 1.31)
Household head				
Male		1		1
Female		0.89 (0.72, 1.11)		0.89 (0.72, 1.11)
Marital status				
Not having a partner		1		1
Had a partner		1.44 (0.71, 2.90)		1.10 (0.54, 2.25)
Religion				
Orthodox		1		1
Muslim		1.52 (1.19, 1.95)		0.93 (0.68, 1.25)
Protestant		2.26 (1.68, 3.02)		1.62 (1.15, 2.28) *
Others		2.21 (1.15, 4.24)		1.48 (0.77, 2.84)
Women education				
Unable to read and write		1		1
Primary education		0.69 (0.57, 0.83)		0.75 (0.62, 0.90) ***
Secondary education		0.39 (0.27, 0.56)		0.50 (0.34, 0.72) ***
Higher education		0.25 (0.13,0.47)		0.34 (0.18, 0.66) ***
Husband education				
Unable to read and write		1		1
Primary education		0.74 (0.61, 0.89)		0.83 (0.69,0.99) *
Secondary education		0.44 (0.53, 0.59)		0.53 (0.39, 0.70) ***
Higher education		0.48 (0.33, 0.69)		0.56 (0.38, 0.81) ***
Women occupation				
Not working		1		1
Working		1.31 (0.72, 2.40)		1.47 (0.80, 2.69)
Husband occupation				
Not working		1		1
Working		0.78 (0.42, 1.42)		0.69 (0.38, 1.26)
Distance to any health facility				
Big problem		1		1
Not a big problem		0.64 (0.54, 0.75)		0.79 (0.66, 0.94) **
Media exposure				
No media exposure		1		1
Has media exposure		0.80 (0.67, 0.59)		0.91 (0.75, 1.11)
Had mobile				
No		1		1
Yes		0.48 (0.38, 0.59)		0.60 (0.48, 0.76) ***
Insurance				
Not insured		1		1
Insured		0.75 (0.32, 1.04)		0.9 (0.88, 1.08)
Had ANC				
No		1		1
Yes		0.14 (0.12, 0.17)		0.18 (0.14, 0.22) ***
Birth order				
1		1		1
2–4		1.76 (1.34, 2.30)		1.78 (1.36, 2.34) ***
> = 5		1.81 (1.24, 2.67)		1.87 (1.27, 2.76) **
Parity				
< = 2		1		1
2–5		1.43 (1.14, 1.81)		1.37 (1.08, 1.74) **
> 5^+^		1.34 (0.93, 1.95)		1.14 (0.78, 1.66)
Wealth index				
Poor		1		1
Middle		0.81 (0.65, 1.02)		0.91 (0.73, 1.14)
Richer		0.48 (0.38, 0.59)		0.78 (0.61, 0.99) *
Residence				
Urban			1	1
Rural			4.55 (3.19, 6.49)	2.92 (1.99, 4.29) ***
Region				
Addis Ababa				1
Tigray			2.54 (1.24, 5.23)	1.46 (0.59, 3.60)
Afar			17.24 (8.06, 6.89)	11.03 (4.29, 28.41) ***
Amhara			8.23 (3.98, 17.00)	6.39 (2.60, 15.70) ***
Oromia			7.07 (3.42, 14.62)	3.69 (1.50, 9.08) **
Somali			10.66 (5.17, 1.98)	6.02 (2.41, 15.06) ***
Benishangul			6.09 (2.90, 12.81)	3.94 (1.59, 9.82) **
SNNPR			5.75 (2.80, 11.77)	3.04 (1.23, 7.47) *
Gambla			6.22 (2.97, 13.02)	4.48 (1.77, 11.32) **
Harari			3.59 (1.69, 7.64)	2.68 (1.04, 6.89) *
Dire Dawa			2.73 (1.28, 5.84)	2.01 (0.77, 5.20)
Community ANC utilization				
Low			1	1
High			0.32 (0.25, 0.41)	0.50 (0.39, 0.65) ***
Community Health facility distance				
Big problem			1	1
Not a big problem			0.56 (0.43, 0.71)	0.69 (0.53, 0.90) **
Community media exposure				
Low			1	1
High			0.85 (0.65, 1.09)	1.11 (0.85, 1.45)
Community poverty status				
High			1	1
Low			0.58 (0.44, 0.75)	0.78 (0.58, 1.04)
Community-women education				
Low			1	1
High			0.55 (0.43, 0.69)	0.78 (0.60, 0.99) *
**Random effects**				
**ICC%**	**67.32**			
**PCV%**	**1**	**82**	**86**	**89**
**MOR**	**12.17**	**4.75**	**3.20**	**2.54**
**Model comparison**				
**Log-likelihood ratio**	**− 4749.96**	**− 2606.26**	**− 4303.85**	**− 2450.68**

NB: * = Significant at *P*-value < 0.05, ** = Significant at *P*-value, 0.01, *** = Significant at *P*-value 0.001, *CI* Confidence Interval, *AOR* Adjusted Odds Ratio

## Discussion

This study revealed that 73.44% (95% CI; 72.60, 74.26) of women deliver at home preceding the survey period. This finding was consistence with the study done Southeast Ethiopia (73.6%) [20], whereas it is higher than the study done at Afar, Ethiopia (65%), South Ethiopia (62.2%), Arba Minch town Ethiopia (33.2%), Akordet town, Eritrea (18%), the urban community at Nigeria (35%), and Ghana (51.2%) [21–26]. The discrepancy might be the study area, setting deference, cultural attitude for health facility delivery, and infrastructure difference (access to the health facility, roads…). In this study, most of the women live in rural areas, which results in a higher prevalence of home delivery. This finding was lower than the 2011 EDHS report (90%), and a study conducted at the rural community of Nigeria (95.3%) [24, 27]. The possible reason might be time difference, and multidimensional strategies are taken to enhance health facility delivery in Ethiopia through health extension workers.

The spatial distribution of home delivery in Ethiopia was non-random in the three successive EDHSs. The spatial distribution of home delivery consistently high in Amhara, and SNNPR regional states of Ethiopia the possible reason might be behavioral, cultural, and health infrastructure inaccessibility. In 2005 EDHS, spatial scan statistics identified the most likely significant cluster at Southwestern Tigray, Amhara, Northern Benishangul Gumuz, and some part of Oromia regional state of Ethiopia. In 2011 and 2016 EDHS most likely significant cluster located at Somali and Oromia regional states of Ethiopia. The possible geographical variation of home delivery in the regions of Ethiopia might be socio-demographic factors, cultural behaviors to health facility delivery, and different infrastructure across the regions of Ethiopia.

Educated women both at the individual and community level were more likely to deliver at the health facility as compared to non-educated women. This finding supported by different studies conducted in Ethiopia [23, 28–30], Sub-Saharan Africa countries, Nepal, and Ghana [26, 31, 32]. Attaining higher education among women can influence the choice of place of delivery in different ways. Educated women would know the benefit of health facility delivery and the danger of giving birth at home through reading newspapers, mass media, and from different social media. Overall, educated women had good health-seeking behavior and the use of health services. Similarly, educated husbands influence the choice of place of delivery, which might be educated husbands would decide on the place of birth for their wives. This finding supported by other studies conducted in Southeast Ethiopia and Ghana [20, 26].

This study evidenced that nearby access to a health facility with a reasonable distance (less than 1 km) positively affects the choice of place of delivery at the individual and community level. Similar findings reported from different studies in Ethiopia, Ghana, and Nigeria [24, 26, 28, 30, 33]. In line with this finding, this study also evidenced that mothers living in a rural area of Ethiopia were more likely to give birth at home than those living in urban, which is also supported by other studies [30, 32, 34]. The possible justification is the fact that mothers living in a rural area far from the health facility. Ethiopia works to expand primary health care facilities through health extension workers at the health post level. Even though accessing services close to the community, still the topography and infrastructure of Ethiopia are difficult to reach for ambulance service. Therefore, mothers living in a rural area didn’t reach the health facility on time for delivery and would influence by the first (mother decision to seek delivery care) and second (reach on time to a health facility) delays for health facility delivery [35].

Another predictor variable that affects the choice of place of delivery was a mobile phone. Women who had a mobile phone or access to mobile phones from their families were 40% less likely to deliver at home than those who had no mobile phone. The number of mobile users and service accessibility increases in Ethiopia [36]. Women having own mobile or the family member can access ambulance on time and service for transportation to the health facility.

Furthermore, women who had at least one antenatal care (ANC) visit at the health facility was 82% less likely to deliver at home than those who had no ANC visit. Similar evidence reported from a meta-analysis in Ethiopia [37], Kenya [38], Nepal [39], and Akordet town, Eritrea [22]. Women during ANC follow up got health education about the choice of place of delivery and the benefit of health facility delivery. Therefore, women during ANC follow up will got behavioral change towards health facility delivery.

The uptake of health facility delivery decreased with high birth order and parity of 2–5, which is consistent with the study conducted in different countries [26, 34, 40, 41]. This might be the service quality given in previous births. Even if the Ethiopian health system has improved in the previous decade, still there were critical shortages of health personnel, inconsistent supplies of drugs and equipment. This could discourage women from utilizing health services in later pregnancies for delivery.

Similar to other studies evidenced [34, 40] that this study revealed that the poor wealth status of a household increases the likelihood of delivering at home. Even though, Ethiopia launches exempted services for delivery service and free transportation of ambulance for laboring mothers still home delivery is an issue. The possible justification might be behavioral reasons and knowledge regarding health facility delivery among the communities.

Furthermore, mothers live in Afar, Amhara, and Somali regional state of Ethiopia highest odds of giving birth at home than in Addis Ababa, which consistence another study has done Ethiopia [13]. This might be the difference in access to health services, infrastructure, and social and cultural attributes. This might be also due to better availability and accessibility of maternal health facilities around Addis Ababa as compared to other regions [13, 42].

This study tried to assess the spatial variation and determinants of home delivery in regions of Ethiopia. Identifying the high-risk area of home delivery in regions of Ethiopia could be used to target intervention for the home delivery reduction in high-risk areas. Therefore, identifying the spatial patterns and determinants of home delivery would help health planners and policymakers in Ethiopia.

### Strength and limitation of the study

This study has strengths of having large dataset include thee EDHS survey and were nationally representative. Multilevel multivariable analysis was used to account for cluster correlations. The spatiotemporal analysis was also used for identifying hotspot areas, most likely clusters and the prediction was performed to predict unsampled/unmeasured areas in the country. However, the limitation of this study is the cross-sectional nature of the study design may affect causality.

## Conclusion

The prevalence of home delivery was 94.78, 90.05, and 73.44% in EDHS 2005, 2011, and 2016 respectively. This study identified a spatiotemporal cluster of home delivery in Amhara and SNNPR regions consistently for Each EDHS year. Women and husband in low education, far from health facility, low wealth status, reside in rural, having a large number of children, uninsured for health, unable to accessing mobile phone, region, low community ANC utilization, low community women education, and big problem distance to any health facility were significant predictors of home delivery in Ethiopia. These results provide further insight in to identifying the true picture of home delivery Spatio-temporal clusters in the country and enable timely spatial targeting factors to alleviate home delivery. Therefore, policymakers and health planners should design an effective intervention program at the identified hot spot areas to reduce home delivery in Ethiopia.

## Data Availability

The data was available from the corresponding author and we can provide upon request.

## Abbreviations

AIC: Akakian Information Criteria
AOR: Adjusted Odds Ratio
ANC: Antenatal Care
EAs: Enumeration Areas
EDHS: Ethiopian Demographic and Health Survey
LLR: Log-Likelihood Ratio
ICC: Intraclass Correlation
MOR: Median Odds Ratio
PCV: Percentage Change in Variance
SDG: Sustainable Development Goal
